# Self-administered version of the Fabry-associated pain questionnaire for adult patients

**DOI:** 10.1186/s13023-015-0325-7

**Published:** 2015-09-17

**Authors:** Barbara Magg, Christoph Riegler, Silke Wiedmann, Peter Heuschmann, Claudia Sommer, Nurcan Üçeyler

**Affiliations:** Department of Neurology, University of Würzburg, Würzburg, Germany; Würzburg Fabry Center for Interdisciplinary Therapy (FAZIT), University of Würzburg, Würzburg, Germany; Institute of Clinical Epidemiology and Biometry, University of Würzburg, Würzburg, Germany; Comprehensive Heart Failure Center, University of Würzburg, Würzburg, Germany; Clinical Trial Center Würzburg, University Hospital Würzburg, Würzburg, Germany

**Keywords:** Fabry disease, Fabry-associated pain, Pain questionnaire

## Abstract

**Background:**

Fabry-associated pain may be the first symptom of Fabry disease (FD) and presents with a unique phenotype including mostly acral burning triggerable pain attacks, evoked pain, pain crises, and permanent pain. We recently developed and validated the first Fabry Pain Questionnaire (FPQ) for adult patients. Here we report on the validation of the self-administered version of the FPQ that no longer requires a face-to-face interview but can be filled in by the patients themselves allowing more flexible data collection.

**Methods:**

At our Würzburg Fabry Center for Interdisciplinary Treatment, Germany, we have developed the self-administered version of the FPQ by adapting the questionnaire to a self-report version. To do this, consecutive Fabry patients with current or past pain history (*n* = 56) were first interviewed face-to-face. Two weeks later patients’ self-reported questionnaire results were collected by mail (*n* = 55). We validated the self-administered version of the FPQ by assessing the inter-rater reliability agreement of scores obtained by supervised administration and self-administration of the FPQ.

**Results:**

The FPQ contains 15 questions on the different pain phenotypes, on pain development during life with and without therapy, and on impairment due to pain. Statistical analysis showed that the majority of questions were answered in high agreement in both sessions with a mean AC1-statistic of 0.857 for 55 nominal-scaled items and a mean ICC of 0.587 for 9 scores.

**Conclusions:**

This self-administered version of the first pain questionnaire for adult Fabry patients is a useful tool to assess Fabry-associated pain without a time-consuming face-to-face interview but via a self-reporting survey allowing more flexible usage.

**Electronic supplementary material:**

The online version of this article (doi:10.1186/s13023-015-0325-7) contains supplementary material, which is available to authorized users.

## Background

The X-linked lysosomal storage disorder Fabry disease (FD) is caused by mutations in the encoding gene of the α-galactosidase A (α-GAL), which lead to reduction or complete loss of enzyme activity. The consequence is the accumulation of the sphingolipid globotriaosylceramide-3 (Gb3) particularly in kidneys, heart, and the nervous system [1]. The only treatment option currently available is enzyme replacement therapy (ERT).

Patients with FD frequently suffer from pain that mostly starts in early childhood [2, 3]. Often small fiber neuropathy is associated with FD and may contribute to Fabry-associated pain [4–8, 3]. Fabry-associated pain is of a distinct phenotype mostly leading to episodic acral burning pain attacks, evoked pain, and pain crises; to a lesser extent permanent pain may be present [3]. We recently developed and validated the first Fabry Pain Questionnaire (FPQ) for adult patients [9]. The FPQ is designed for a face-to-face interview and covers questions on Fabry specific pain characteristics. To allow pain assessment in Fabry patients without personal report at a referral Fabry Center we developed and validated the self-reporting version of the FPQ.

## Methods

### Development of the self-administered version of the FPQ

Our study was approved by the Ethics Committee of the Medical Faculty of the University of Würzburg (#25/13), and written informed consent was obtained before inclusion from every study participant. On the basis of the recently validated face-to-face version of the FPQ [9] we first adapted the introductory parts of its 15 questions to a more explanatory version (NÜ, BM, CS) allowing patients to understand and answer the questions without the oral instructions of an interviewer. This first version was reviewed and revised (SW, PH) before the self-administered FPQ (saFPQ) was then validated. In brief, the FPQ assesses the four Fabry-associated pain phenotypes (pain attacks, evoked pain, pain crises, permanent pain) in childhood and adulthood with regard to pain presence, localization (including a pain drawing), frequency, qualities, triggers, and development over time with and without ERT and symptomatic treatment. Additionally, patients are asked about pain-associated impairment at work and during everyday life.

### Study design

From July 2014 to February 2015 study participants were first interviewed face-to-face with the saFPQ by a trained interviewer (BM) during their visit at our Fabry Center for Interdisciplinary Treatment (FAZIT) or were visited at their homes. The questions were read out to the patient, and the individual answers and the time necessary to complete the questionnaire were noted. Patients were then asked to individually fill in the questionnaire again two weeks later at their homes and to send back the questionnaire by mail. Patients also noted the time needed to complete the questionnaire.

### Patients

The following inclusion criteria were applied for the study cohort: ≥18 year old men or women with genetically proven FD; current and/or past pain history. Patients were recruited at FAZIT in Germany, which is a tertiary referral center. Patients had previously not taken part in the evaluation of the FPQ [9].

### Statistical analysis

All analyses were performed using IBM SPSS Version 22 (Ehningen, Germany) and SAS, version 9.3 (SAS Institute Inc, Cary, NC, USA). According to previous studies [10] and with regard to the number of patients seen at FAZIT, the number of available and suitable cases was calculated. Of the approximately 220 Fabry patients known at our Center 60-70 % of patients do and/or did suffer from pain in adulthood and/or childhood. Of these patients 62 had already been interviewed during the validation of the face-to-face version of the FPQ [9]. In order to control for the influence of current pain intensity on how patients answered the questions in the two sessions, we *a priori* defined that analyses would be restricted to patients who reported identical current pain intensities at both sessions, defined as a deviation of ≤1 point on a numeric rating scale (NRS) for question 6 of the FPQ. For a detailed description of the statistical analysis performed see Additional file 1.

## Results

### Study cohort

Table 1 summarizes demographic data of the study participants. Fifty-six Fabry patients of the FAZIT fulfilling the inclusion criteria were enrolled. Of these, 55 patients received a first face-to-face interview and two weeks later filled in and sent back the saFPQ. One patient did not fill in and/or send back the questionnaire and was lost to follow-up. 15 patients were excluded because they reported varying current pain intensities on question 6 of the FPQ. Finally, the study cohort consisted of 40 Fabry patients (22 men, 18 women; median age: 42 years, range 21 to 67 years). None of the patients reported pain only in childhood while 34/40 (85 %) patients had pain in childhood and adulthood; 6/40 (15 %) patients reported pain only in adulthood. With regard to the current pain phenotypes, 36/40 (90 %) patients reported pain attacks, 32/40 (80 %) had pain crises, 27/40 (68 %) suffered from evoked pain, and 18/40 (45 %) had permanent pain.

**Table 1:** Demographic data of the study population

Number of patients (N)	40
M, F (N)	22, 18
Median age (range)	42 (21–67)
Pain only in childhood (M/F)	none
Pain only in adulthood (M/F)	6 (0/6)
Pain in childhood and adulthood (M/F)	34 (22/12)

Shows demographic data of the study population and numbers of patients with pain only in childhood/adulthood or in childhood as well as in adulthood. M = Male, F = Female

### Face-validity

All saFPQ items were answered completely by all study participants. The overall acceptance by the patients was high and patients gave a very positive feedback about the content and the format of the questions. The median time necessary to fill in the saFPQ during the face-to-face interview was 15 min (range 10–20 min), and during the self-filling-in period 15 min (range 10–30 min).

### Assessing inter-rater reliability for the self-administered and the face-to-face version

Agreement between the self-administered and the face-to-face version was very high for nominal-scaled items with a mean of all AC1-statistics of 0.857 (range 0.580-1.000). With the exception of evoked pain by contact (4a), all items of questions 1–5 (referring to the four major pain phenotypes and sensitivity impairment) showed good agreement with AC1-statistics (≥0.600; Table 2). Question 8 (pain location) had very good agreement with AC1-statistics (≥0.800 for all items; Table 2). Assessing agreement of question 10 (last pain event), we had the problem that most of the patients had one or more pain events between the two sessions of interview/self-report and therefore answered the question differently. Thus, we only analyzed 11 patients with no pain in the meantime, with a good AC1-statistics result (Table 2). Pain quality reported by patients in question 11 showed high agreement across all items, with only two AC1-statistics between 0.600 and 0.800 and the other ones having values of ≥0.800 (Table 2). Results for questions 12 (pain triggers) and 13 (impairment at work due to pain) also showed high agreement (Table 2). Regarding the scored items of the questionnaire, questions 7a and 7b (pain frequency) had poor inter-rater reliability, while the other questions reached good inter-rater reliability with ICCs from 0.577 to 0.970 (Table 2). For question 7b, all patients without ERT were excluded from analysis with 22 patients remaining. The mean ICC for the interval-scaled items was 0.587. No assessment of agreement was performed for free text questions 2a, 3a and 9. The final German version of the saFPQ is provided in the supplement section (Additional file 2). A first, not yet formally validated English translation is also added (Additional file 3).

**Table 2:** Results of statistical analysis on test-retest reliability of the FPQ questions

saFPQ question	AC1-statistic (95 % confidence interval)	Scale	Statistics
1) Do you have permanent pain in adulthood or did you have permanent pain in childhood?	Adulthood: 0.901 (0.766–1)	Nominal	AC1
	Childhood: 0.834 (0.697–0.971)		
2) Do you have pain attacks in adulthood or did you have pain attacks in childhood?	Adulthood: 0.939 (0.855–1)	Nominal	AC1
	Childhood: 0.875 (0.757–0.993)		
2a) If you have pain attacks in adulthood or if you had pain attacks in childhood: how often did/how often do these pain attacks occur and for how long did/how long do these pain attacks last?	No statistics		
3) Do you have pain crises in adulthood or did you have pain crises in childhood?	Adulthood: 0.714 (0.520–0.908)	Nominal	AC1
	Childhood: 0.801 (0.652–0.950)		
3a) If you have pain crisis in adulthood or if you had pain crisis in childhood: how frequent were/are these pain crises and how long did/do they last in average?	No statistics		
4a) Do you have in adulthood or did you have in childhood pain that can be triggered by touch?	Adulthood: 0.593 (0.356–0.830)	Nominal	AC1
	Childhood: 0.580 (0.390–0.770)		
4b) Do you have in adulthood or did you have in childhood pain that can be triggered by a cold object?	Adulthood: 0.730 (0.559–0.901)	Nominal	AC1
	Childhood: 0.688 (0.506–0.870)		
4c) Do you have in adulthood or did you have in childhood pain that can be triggered by a warm object?	Adulthood: 0.800 (0.651–0.949)	Nominal	AC1
	Childhood: 0.663 (0.479–0.847)		
4d) Do you have in adulthood or did you have in childhood pain that can be triggered by pressure?	Adulthood: 0.817 (0.645–0.989)	Nominal	AC1
	Childhood: 0.767 (0.608–0.926)		
5) Do you have in adulthood or did you have in childhood sensory impairment like numbness or tingling in the painful body area?	Adulthood: 0.742 (0.579–0.905)	Nominal	AC1
	Childhood: 0.688 (0.517–0.859)		
6) What is your pain intensity at the moment?^a^	0.970 (0.945–0.984)	Interval	ICC
7) How did your pain develop over time (with or without treatment)?	Frequency: 0.400 (0.103–0.632)	Interval	ICC
7a) Since last visit in Würzburg	Intensity: 0.173 (−0.145–0.459)		
7) How did your pain develop over time (with or without treatment)?	Frequency: 0.123 (−0.299–0.508)	Interval	ICC
7b) Under enzyme replacement therapy	Intensity: 0.577 (0.222–0.798)		
7) How did your pain develop over time (with or without treatment)?	Frequency: 0.902 (0.819–0.948)	Interval	ICC
7c) During life	Intensity: 0.764 (0.592–0.869)		
8) Please indicate the body areas that are mainly affected when you have pain.	Hands: 0.939 (0.857–1)	Nominal	AC1
	Feet: 0.906 (0.802–1)		
	Back/neck: 0.836 (0.701–0.971)		
	Knees: 1 (−)		
	Shoulders: 0.892 (0.772–1)		
	Other joints: 0.897 (0.783–1)		
	Abdomen/thorax: 0.859 (0.726–0.992)		
	Head/jaws: 0.878 (0.743–1)		
	Arms/legs: 0.804 (0.641–0.967)		
9) Which analgesic drugs do you take?	No statistics		
10) When was the last time you had pain?	No statistics		
10a) What type of pain was your last pain?	0.889 (0.671–1)	Nominal	AC1
10b) The last time you had pain: what was its maximum intensity on a scale from zero to ten?	0.875 (0.630–1)	Nominal	AC1
10c) The last time you had pain: what was its average intensity on a scale from zero to ten?	0.620 (0.236–1)	Nominal	AC1
11) How does your pain feel?	Adulthood burning: 0.902 (0.794–1)	Nominal	AC1
	Childhood burning: 0.846 (0.701–0.991)		
	Adulthood stabbing: 0.754 (0.550–0.958)		
	Childhood stabbing: 0.800 (0.612–0.988)		
	Adulthood pulling: 0.853 (0.714–0.992)		
	Childhood pulling: 0.902 (0.420–0.867)		
	Adulthood like electric shocks: 0.644 (0.420–0.867)		
	Childhood like electric shocks: 0.840 (0.689–0.991)		
	Adulthood tearing: 0.915 (0.821–1)		
	Childhood tearing: 0.919 (0.831–1)		
	Adulthood don’t know: 1 (−)		
	Childhood don’t know: 0.945 (0.871–1)		
12) Are there triggers for your pain?	Adulthood without trigger: 0.751 (0.545–0.957)	Nominal	AC1
	Childhood without trigger: 0.873 (0.734–1)		
	Adulthood heat: 0.917 (0.803–1)		
	Childhood heat: 0.951 (0.855–1)		
	Adulthood cold: 0.901 (0.766–1)		
	Childhood cold: 0.864 (0.715–1)		
	Adulthood fever: 1 (−)		
	Childhood fever: 1 (−)		
	Adulthood physical activity: 0.877 (0.742–1)		
	Childhood physical activity: 0.95 (0.854–1)		
	Adulthood sports: 1 (−)		
	Childhood sports: 1 (−)		
	Adulthood don’t know: 1 (−)		
	Childhood don’t know: 1 (−)		
13) How many days without work (including housework) did you have in the last year due to pain?	0.967 (0.902–1)	Nominal	AC1
14) How much does pain influence your working ability (including housework) in general on a scale from zero to ten?	0.693 (0.491–0.825)	Interval	ICC
15) How much does pain influence your leisure activities in general on a scale from zero to ten?	0.680 (0.472–0.816)	Interval	ICC

Table 2 gives details on data scales and the statistics used for each saFPQ item

AC1, ICC, *saFPQ* self-administered Fabry Pain Questionnaire

^a^Only patients with a difference ≤1 point included in analysis

## Discussion

We present the self-administered version of the first pain questionnaire for adult Fabry patients which is based on the published face-to-face interview version [9]. This self-administered version allows pain assessment and follow-up also in patients that are not able to personally come to their referral Fabry Center. Additionally, the saFPQ reduces evaluation time of the treating physician in clinical practice due to its self-reporting nature.

Recently, a pain questionnaire as part of a data collection on Fabry symptoms and items was published to be used in children with FD [11]. FPQ was the first pain questionnaire for adult Fabry patients [9] covering all relevant items of this very special pain phenotype instead of asking about few and selected pain characteristics only [12]. Based on our longstanding experience with Fabry-associated pain at one of the largest German Fabry centers, FPQ helps obtaining data on current and childhood Fabry-associated pain in a standardized manner [9]; it fills a gap where standardized pain questionnaires fail to properly reflect Fabry-associated pain [13, 3, 14].

Since its publication FPQ has been used routinely at the FAZIT allowing comprehensive patient assessment and data collection. Patients’ feedback is very positive and further German Fabry Centers have started applying FPQ in their clinical routine. Particularly, the inclusion of the FPQ as an assessment tool for data collection in multi-center and large scale studies and in Fabry registries is warranted (personal communication). However, in its original version with a face-to-face interview design the FPQ was unpractical and time-consuming for the treating physicians. To overcome this drawback we have now designed and validated the self-administered version of the FPQ that allows the assessment of Fabry patients in a self-reporting manner and even if they cannot personally come to the referral Fabry Center.

One limitation of our study is the relatively low number of subjects, however, the statistical analysis of the saFPQ showed that the majority of questions have a good to very good test-retest-reliability. Few questions were obviously difficult to answer in the study setting with two time points and potential events in between like question 7: “How did your pain (with or without treatment) develop since last visit ?” Some patient referred to the last visit two weeks ago (i.e., to the time point of the face-to-face interview) and some patients referred to their last regular visit at the FAZIT one year ago. This, however, is due to the special study situation with two consecutive interviews, which is not clinical routine and therefore without practical relevance. One reason why some patients may have given inconsistent answers to some of the questions at the two sessions may be that particularly patients with low-frequent episodic pain do not see this (manageable) pain as a major problem and therefore judge changes less consistently when asked at two different time points. Another reason for the inconsistent answers particularly with regard to the analgesic effect of ERT may be based on the experiences patients made during ERT supply shortage (http://www.fda.gov/downloads/Drugs/DrugSafety/DrugShortages/UCM187056.pdf). During this period not only ERT dosage but also the compound (agalsidase-beta switch to agalsidase-alpha) was changed in some patients. It may have been difficult for these patients to judge if any changes in their pain during this phase were due to changes in ERT dosage or compound, or whether this was part of the natural course of their disease. Another aspect that needs to be taken into account are the known alterations in cognitive function of Fabry patients [15]. These may influence consistent replies on repetitively asked questions, which is also a known phenomenon in clinical routine e.g., during history taking. Since the main aim of our study was to investigate questionnaire reliability, we did not compare our questionnaire with other tools such as the Brief Pain Inventory (BPI). The BPI does not contain Fabry-associated questions, but has been applied in large cohorts of FD patients e.g., in the Fabry registries.

In the setting of a rare disease, it is not feasible that patients travel to the referral Fabry Center for any change in symptoms. To still be able to obtain standardized data, the saFPQ will be a useful tool. This may be of particular relevance during clinical trials when patients need to apply self-report instruments between visits. Now pain data from Fabry patients (for clinical practice and trials) can even be obtained in remote areas where only few or no Fabry Centers are present. Thus, saFPQ brings further flexibility to the assessment of Fabry-associated pain. The next step will be to validate the English version of the saFPQ to make the questionnaire available for even more Fabry patients.

## Conclusion

Fabry-associated pain is distinct in phenotype and needs special tools for standardized assessment during clinical practice and in trials. The saFPQ is the first self-administered pain questionnaire designed for Fabry patients and will substantially improve pain management and data acquisition in patients suffering from Fabry-associated pain.

## Additional files

Additional file 1:
**Supplementary methods.** (DOC 40 kb) Additional file 2:
**Original and validated German version of the self-administered Fabry Pain Questionnaire.** (DOC 402 kb) Additional file 3:
**Not yet validated English translation of the self-administered Fabry Pain Questionnaire.** (DOC 275 kb)

## References

[1] Toyooka K (2011). Fabry disease. Curr Opin Neurol.

[2] Burlina AP, Sims KB, Politei JM, Bennett GJ, Baron R, Sommer C (2011). Early diagnosis of peripheral nervous system involvement in Fabry disease and treatment of neuropathic pain: the report of an expert panel. BMC Neurol.

[3] Üçeyler N, Ganendiran S, Kramer D, Sommer C (2014). Characterization of pain in fabry disease. Clin J Pain.

[4] Liguori R, Di Stasi V, Bugiardini E, Mignani R, Burlina A, Borsini W (2010). Small fiber neuropathy in female patients with fabry disease. Muscle Nerve.

[5] Laaksonen SM, Roytta M, Jaaskelainen SK, Kantola I, Penttinen M, Falck B (2008). Neuropathic symptoms and findings in women with Fabry disease. Clin Neurophysiol.

[6] Torvin Moller A, Winther Bach F, Feldt-Rasmussen U, Rasmussen A, Hasholt L, Lan H (2009). Functional and structural nerve fiber findings in heterozygote patients with Fabry disease. Pain.

[7] Biegstraaten M, Binder A, Maag R, Hollak CE, Baron R, van Schaik IN (2011). The relation between small nerve fibre function, age, disease severity and pain in Fabry disease. Eur J Pain.

[8] Üçeyler N, He L, Schönfeld D, Kahn AK, Reiners K, Hilz MJ (2011). Small fibers in Fabry disease: baseline and follow-up data under enzyme replacement therapy. J Peripher Nerv Syst.

[9] Üçeyler N, Magg B, Thomas P, Wiedmann S, Heuschmann P, Sommer C (2014). A comprehensive Fabry-related pain questionnaire for adult patients. Pain.

[10] Nolte CH, Malzahn U, Rakow A, Grieve AP, Wolfe CD, Endres M (2010). The German version of the satisfaction with stroke care questionnaire (SASC) for stroke patients. Fortschr Neurol Psychiatr.

[11] Ramaswami U, Stull DE, Parini R, Pintos-Morell G, Whybra C, Kalkum G (2012). Measuring patient experiences in Fabry disease: validation of the Fabry-specific Pediatric Health and Pain Questionnaire (FPHPQ). Health Qual Life Outcomes.

[12] Gibas AL, Klatt R, Johnson J, Clarke JT, Katz J (2006). A survey of the pain experienced by males and females with Fabry disease. Pain Res Manag.

[13] Bouhassira D, Attal N, Fermanian J, Alchaar H, Gautron M, Masquelier E (2004). Development and validation of the Neuropathic Pain Symptom Inventory. Pain.

[14] Von Korff M, Ormel J, Keefe FJ, Dworkin SF (1992). Grading the severity of chronic pain. Pain.

[15] Bolsover FE, Murphy E, Cipolotti L, Werring DJ, Lachmann RH (2014). Cognitive dysfunction and depression in Fabry disease: a systematic review. J Inherit Metab Dis.

