# Supplementary material for: Self-administered version of the Fabry-associated pain questionnaire for adult patients
Source: Orphanet J Rare Dis. 2015 Sep 17;10:113. doi: 10.1186/s13023-015-0325-7 (PMC4573689; doi:10.1186/s13023-015-0325-7)
Supplement: Additional file 1: — Supplementary methods. (DOC 40 kb) [file 13023_2015_325_MOESM1_ESM.doc]

**Additional file 1**

**Statistical analysis**

For assessing the agreement of nominally scaled items of the questionnaire, we decided not to use kappa-coefficients because of its well-known weaknesses, but to use Gwet’s AC1-statistics as an advanced and more appropriate method . Firstly, kappa is not particularly a “chance-corrected measure of agreement” because the proportion of chance agreement is calculated assuming that the rater decisions are stochastically independent, which is, however, an improper assumption. Secondly, the kappa statistic is very sensitive to the rater’s classification probabilities and to the respective category`s prevalence in the subject population . Gwet’s AC1-statistic was used to overcome these weaknesses. AC1-statistics between 0 and 0.2 were assessed as very low, between 0.2 and 0.4 as low, between 0.4 and 0.6 as adequate, between 0.6 and 0.8 as good, and between 0.8 and 1.0 as very good. The same definition was used for the ICC. For assessing the inter-rater reliability of the two questionnaires (method one, face-to-face FPQ and method two, pFPQ) for scored items, analyses were conducted via Intraclass-Correlation-Coefficients (ICCs), which were calculated as previously described . For question 10 with only 11 evaluable patients (see results), we recoded the original NRS entries with values from 0 to 10 as categorical dummy variables with 4 categories (0=no pain, 1-3=mild pain, 4-7=intermediate pain, 8-10=severe pain) and assessed agreement via AC1-statistics. In order to assess agreement of questions 8, 11 and 12, dummy variables (yes/no) were created to test agreement individually for every pain location, pain quality, and pain triggers via AC1-statistics. Agreement of question 13 (missing working days during last year including household), was assessed via AC1-statistics after creating a dummy variable with 3 categories (0 days, ≤20 days, ≥20 days).

**References:**

1. Gwet KL. Computing inter-rater reliability and its variance in the presence of high agreement. Br J Math Stat Psychol. 2008;61:29-48.

2. Nolte CH, Malzahn U, Rakow A, Grieve AP, Wolfe CD, Endres M et al. [The German version of the satisfaction with stroke care questionnaire (SASC) for stroke patients]. Fortschr Neurol Psychiatr. 2010;78:355-9.

3. Üçeyler N, Magg B, Thomas P, Wiedmann S, Heuschmann P, Sommer C. A comprehensive Fabry-related pain questionnaire for adult patients. Pain. 2014;155:2301-5.

4. Shrout PE, Fleiss JL. Intraclass correlations: uses in assessing rater reliability. Psychol Bull. 1979;86:420-8.
