# Supplementary material for: Self-administered version of the Fabry-associated pain questionnaire for adult patients
Source: Orphanet J Rare Dis. 2015 Sep 17;10:113. doi: 10.1186/s13023-015-0325-7 (PMC4573689; doi:10.1186/s13023-015-0325-7)
Supplement: Additional file 2: — Original and validated German version of the self-administered Fabry Pain Questionnaire. (DOC 402 kb) [file 13023_2015_325_MOESM2_ESM.doc]

# Additional file 2

# Selbstausfüller Version des Würzburger Fabry Schmerzfragebogens (saW-FSF)

# Datum: _______________________________________________________

Name: _______________________________________________________

Geburtsdatum: ________________________________________________

| Liebe Patientin, Lieber PatientMit den nachfolgenden Fragen möchten wir erfassen, ob Sie unter Schmerzen leiden, die typisch für den M. Fabry sind. Wir möchten erfahren, wie Ihre Schmerzen ausgeprägt sind und wie sehr sie Sie in Ihrem Alltagsleben beeinträchtigen. Vielleicht treffen nicht alle der folgenden Fragen auf Sie zu. Bitte beantworten Sie trotzdem ALLE Fragen. Bitte beachten Sie, dass sich ein Teil der Fragen auch auf Ihre Kindheit (d.h. bis zum 18. Lebensjahr) bezieht. Nicht alle Beispiele werden genau auf Ihren Schmerz passen. Versuchen Sie bitte trotzdem, sich ähnliche Situationen in Ihrem Leben vorzustellen und alle Fragen zu beantworten. |
| --- |

| **1) Haben Sie als Erwachsener oder hatten Sie als Kind Dauerschmerzen?**  Dauerschmerzen sind Schmerzen jeglicher Stärke (leicht bis unerträglich stark), die 24 Stunden oder die überwiegende Zeit eines Tages anhalten. | | |
| --- | --- | --- |
|  | Als Erwachsener | In der Kindheit |
| Ja | O | O |
| Nein | O | O |
| Weiß nicht | O | O |

| **2) Haben Sie als Erwachsener oder hatten Sie als Kind Schmerzattacken?**  Schmerzattacken sind Schmerzen jeglicher Stärke (leicht bis unerträglich stark), die mit oder ohne Auslöser plötzlich einsetzen, für eine bestimmte Zeit anhalten und dann wieder vergehen. | | |
| --- | --- | --- |
|  | Als Erwachsener | In der Kindheit |
| Ja | O | O |
| Nein | O | O |
| Weiß nicht | O | O |

| **2a) Wenn Sie als Erwachsener oder als Kind Schmerzattacken haben bzw. hatten: wie häufig treten bzw. traten diese auf und wie lange dauern bzw. dauerten diese Schmerzen an?** | | |
| --- | --- | --- |
|  | Als Erwachsener | In der Kindheit |
| Häufigkeit  (z.B. zweimal im Monat) |  |  |
| Dauer  (z.B. 1 Stunde) |  |  |
| Weiß nicht | O | O |

| **3) Haben Sie als Erwachsener oder hatten Sie als Kind Schmerzkrisen?**  Schmerzkrisen sind massive Schmerzen, die Teile des Körpers oder den ganzen Körper betreffen können und die bis zu mehrere Tage anhalten können (Beispiel: Massive Schmerzen während eines fieberhaften Infekts). | | |
| --- | --- | --- |
|  | Als Erwachsener | In der Kindheit |
| Ja | O | O |
| Nein, dann weiter mit 4a | O | O |
| Weiß nicht | O | O |

| **3a) Wenn Sie als Erwachsener oder als Kind Schmerzkrisen haben bzw. hatten: wie häufig treten bzw. traten diese auf und wie lange dauern bzw. dauerten diese Schmerzen im Durchschnitt an?** | | |
| --- | --- | --- |
|  | Als Erwachsener | In der Kindheit |
| Häufigkeit  (z.B. zweimal im Monat) |  |  |
| Dauer  (z.B. 1 Tag) |  |  |
| Weiß nicht | O | O |

| **4a) Haben Sie als Erwachsener oder hatten Sie als Kind Schmerzen, die durch Berührung ausgelöst werden können?**  (Beispiel: Wenn Sie im Bad barfuß über Fließen laufen) | | |
| --- | --- | --- |
|  | Als Erwachsener | In der Kindheit |
| Ja | O | O |
| Nein | O | O |
| Weiß nicht | O | O |

| **4b) Haben Sie als Erwachsener oder hatten Sie als Kind Schmerzen, die durch einen kalten Gegenstand ausgelöst werden können?**  (Beispiel: Wenn Sie im Winter das kalte Lenkrad anfassen) | | |
| --- | --- | --- |
|  | Als Erwachsener | In der Kindheit |
| Ja | O | O |
| Nein | O | O |
| Weiß nicht | O | O |

| **4c) Haben Sie als Erwachsener oder hatten Sie als Kind Schmerzen, die durch einen warmen Gegenstand ausgelöst werden können?**  (Beispiel: Wenn Sie einen warmen Teller aus der Spülmaschine nehmen) | | |
| --- | --- | --- |
|  | Als Erwachsener | In der Kindheit |
| Ja | O | O |
| Nein | O | O |
| Weiß nicht | O | O |

| **4d) Haben Sie als Erwachsener oder hatten Sie als Kind Schmerzen, die durch Druck ausgelöst werden können?**  (Beispiel: Schmerzen, die auftreten, wenn Sie einen engen Schuh anhaben und verschwinden, wenn Sie diesen ausziehen) | | |
| --- | --- | --- |
|  | Als Erwachsener | In der Kindheit |
| Ja | O | O |
| Nein | O | O |
| Weiß nicht | O | O |
| **5) Haben Sie als Erwachsener oder hatten Sie als Kind in den schmerzenden Körperbereichen Gefühlsstörungen im Sinn von Taubheitsgefühl oder Kribbeln?** (Taubheitsgefühl = das Gefühl, das man erlebt, wenn ein Fuß „einschläft“;  Kribbeln = das Gefühl wie viele kleine Nadelstiche, wenn der Fuß wieder „aufwacht“) | | |
|  | Als Erwachsener | In der Kindheit |
| Nein | O | O |
| Taubheitsgefühl | O | O |
| Kribbeln | O | O |
| Weiß nicht | O | O |

| **6) Bitte geben Sie auf einer Skala von null bis zehn an, wie stark Ihr Schmerz jetzt im Augenblick ist. Null bedeutet dabei „kein Schmerz“ und zehn „stärkster vorstellbarer Schmerz“.** | |
| --- | --- |
|  | Bitte nur eine Zahl ankreuzen |
| 0 – 1 – 2 – 3 – 4 – 5 – 6 – 7 – 8 – 9 – 10  kein stärkster  Schmerz vorstellbarer Schmerz | |

| **7) Wie haben sich Ihre Schmerzen (mit oder ohne Therapie) entwickelt?** | |
| --- | --- |
| **a) seit der letzten Vorstellung in Würzburg**  Bitte kreuzen Sie auf den nachfolgenden Zahlenstreifen jeweils die für Sie zutreffende Zahl an. Die Zahlenstreifen gehen von -10 bis 10. Bei der Schmerzhäufigkeit bedeutet -10, dass Sie sehr viel seltener an Schmerzen leiden; null, dass sich nichts geändert hat; 10, dass Sie sehr viel häufiger an Schmerzen leiden. | |
|  | Bitte nur eine Zahl ankreuzen |
| **HINSICHTLICH HÄUFIGKEIT:**  -10 -9 -8 -7 -6 -5 -4 -3 -2 -1 0 1 2 3 4 5 6 7 8 9 10  Schmerz seltener als zuvor  Schmerz häufiger als zuvor Ich kann dazu keine Angaben machen: O **HINSICHTLICH STÄRKE:**  -10 -9 -8 -7 -6 -5 -4 -3 -2 -1 0 1 2 3 4 5 6 7 8 9 10  Schmerz weniger stark als zuvor  Schmerz stärker als zuvor Ich kann dazu keine Angaben machen: O | |
| **b) unter Enzymersatztherapie:**  Bitte kreuzen Sie auf den nachfolgenden Zahlenstreifen jeweils die für Sie zutreffende Zahl an. Die Zahlenstreifen gehen von -10 bis 10. Bei der Schmerzhäufigkeit bedeutet -10, dass Sie sehr viel seltener an Schmerzen leiden; null, dass sich nichts geändert hat; 10, dass sie sehr viel häufiger an Schmerzen leiden. Falls Sie keine Enzymersatztherapie erhalten, kreuzen Sie bitte „Ich kann dazu keine Angaben machen“ an. | |
|  | Bitte nur eine Zahl ankreuzen |
| **HINSICHTLICH HÄUFIGKEIT:**  -10 -9 -8 -7 -6 -5 -4 -3 -2 -1 0 1 2 3 4 5 6 7 8 9 10  Schmerz seltener als vor Enzymersatztherapie  Schmerz häufiger als vor Enzymersatztherapie Ich kann dazu keine Angaben machen: O **HINSICHTLICH STÄRKE:**  -10 -9 -8 -7 -6 -5 -4 -3 -2 -1 0 1 2 3 4 5 6 7 8 9 10  Schmerz weniger stark als vor Enzymersatztherapie  Schmerz stärker als vor Enzymersatztherapie Ich kann dazu keine Angaben machen: O | |
| **c) im Verlauf Ihres Lebens (von ihrer Geburt bis zum jetzigen Zeitpunkt):**  Bitte kreuzen Sie auf den nachfolgenden Zahlenstreifen jeweils die für Sie zutreffende Zahl an. Die Zahlenstreifen gehen von -10 bis 10. Bei der Schmerzhäufigkeit bedeutet -10, dass Sie sehr viel seltener an Schmerzen leiden; null, dass sich nichts geändert hat; 10, dass Sie sehr viel häufiger an Schmerzen leiden. | |
|  | Bitte nur eine Zahl ankreuzen |
| **HINSICHTLICH HÄUFIGKEIT:**  -10 -9 -8 -7 -6 -5 -4 -3 -2 -1 0 1 2 3 4 5 6 7 8 9 10  Schmerz seltener als früher  Schmerz häufiger als früher Ich kann dazu keine Angaben machen: O **HINSICHTLICH STÄRKE:**  -10 -9 -8 -7 -6 -5 -4 -3 -2 -1 0 1 2 3 4 5 6 7 8 9 10  Schmerz weniger stark als früher  Schmerz stärker als früher Ich kann dazu keine Angaben machen: O | |

| **8) Bitte zeichnen Sie ein, welche Körperstellen hauptsächlich betroffen sind, wenn Sie Schmerzen haben.** |
| --- |


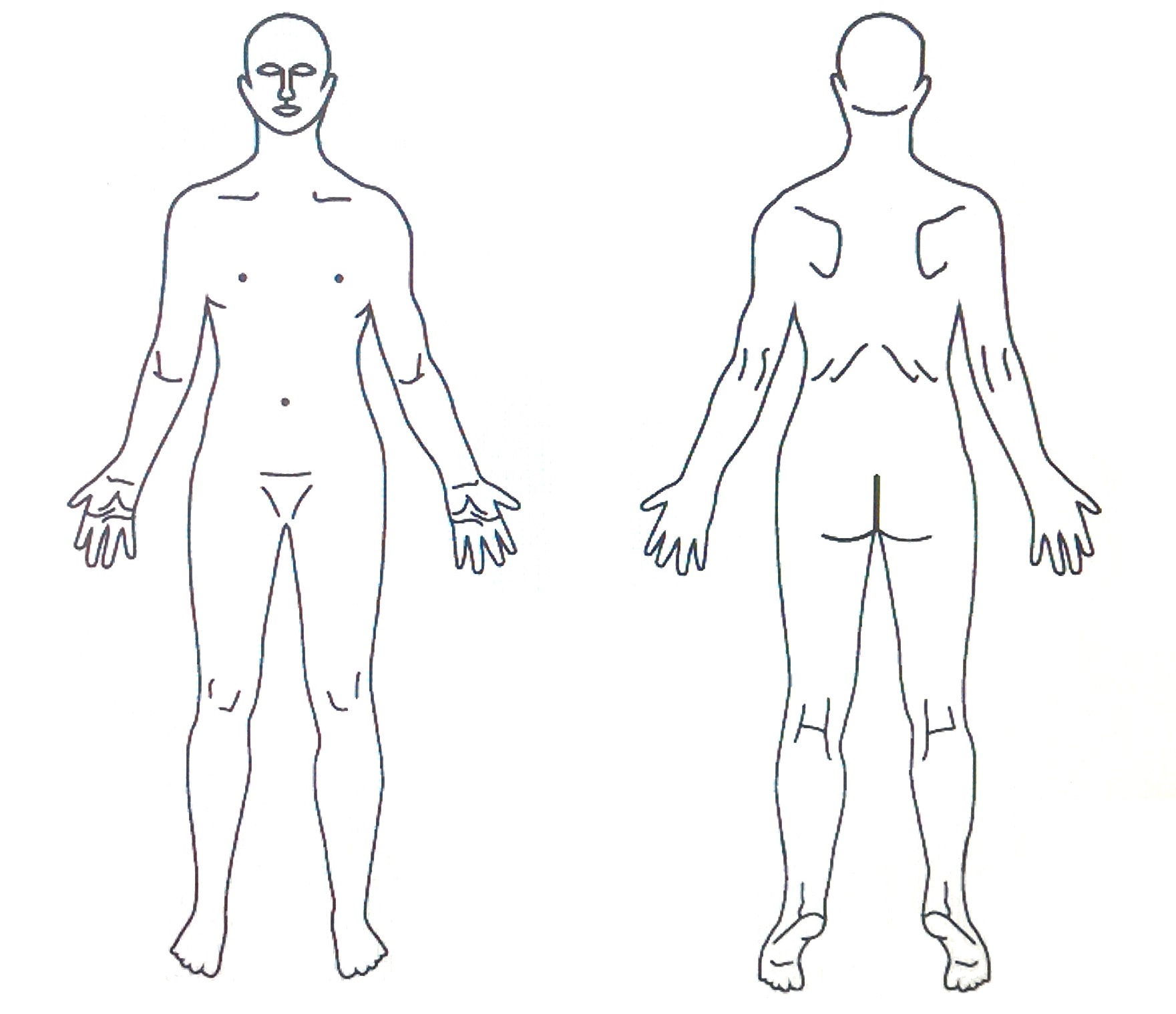


| **9) Welche Schmerzmedikamente nehmen Sie ein? Bitte tragen Sie in nachfolgende Tabelle das Präparat, die Dosis und die Einnahmehäufigkeit pro Tag an.** | |
| --- | --- |
| Ich nehme keine Schmerzmedikamente ein | O |
| Ich nehme folgende Medikamente gegen Schmerzen ein:   | **Präparat** | **Dosis** | **Tägliche Einnahme** | | --- | --- | --- | | Beispiel: Pregabalin | 75 mg | 1-0-1 | | Beispiel: Paracetamol | 500 mg | Bei Bedarf | |  |  |  | |  |  |  | |  |  |  | |  |  |  | |  |  |  | |  |  |  | |  |  |  | | |

| **10) Wann hatten Sie zuletzt Schmerzen? Bitte geben Sie den Zeitpunkt so genau wie möglich an. Wenn Sie sich nicht genau erinnern können geben Sie bitte den ungefähren Zeitraum an.** |
| --- |
|  |

| **10a) Welche Art von Schmerz war das?** | |
| --- | --- |
|  | **Sie können mehrere Kreise ankreuzen** |
| **Dauerschmerz**  (Dauerschmerzen sind Schmerzen jeglicher Stärke, leicht bis unerträglich stark, die 24 Stunden oder die überwiegende Zeit eines Tages anhalten) | O |
| **Schmerzattacke**  (Schmerzattacken sind Schmerzen jeglicher Stärke (leicht bis unerträglich stark), die mit oder ohne Auslöser plötzlich einsetzen, für eine bestimmte Zeit anhalten und dann wieder vergehen) | O |
| **Schmerzkrise**  (Schmerzkrisen sind massive Schmerzen, die Teile des Körpers oder den ganzen Körper betreffen können und die bis zu mehrere Tage anhalten können) | O |
| **Durch Berührung von nicht-schmerzhaften Reizen ausgelöste Schmerzen** | O |
| Andere, und zwar: |  |
| Weiß nicht | O |

| **10b) Als Sie zuletzt Schmerzen hatten: Wie stark war die stärkste Ausprägung Ihres Schmerzes auf einer Skala von null bis zehn? Null bedeutet dabei „kein Schmerz“ und zehn „stärkster vorstellbarer Schmerz“.** | |
| --- | --- |
|  | Bitte nur eine Zahl ankreuzen |
| 0 – 1 – 2 – 3 – 4 – 5 – 6 – 7 – 8 – 9 – 10  kein stärkster  Schmerz vorstellbarer SchmerzKeine Angabe o | |

| **10c) Als Sie zuletzt Schmerzen hatten: Wie stark war ihre Schmerzstärke im Durchschnitt auf einer Skala von null bis zehn? Null bedeutet dabei „kein Schmerz“ und zehn „stärkster vorstellbarer Schmerz“.** | |
| --- | --- |
|  | Bitte nur eine Zahl ankreuzen |
| 0 – 1 – 2 – 3 – 4 – 5 – 6 – 7 – 8 – 9 – 10  kein stärkster  Schmerz vorstellbarer Keine Angabe o Schmerz | |

| **11) Wie fühlt sich Ihr Schmerz an? Hier können Sie mehrere Antworten ankreuzen.** | | | | |
| --- | --- | --- | --- | --- |
|  | | Als Erwachsener | In der Kindheit | |
| brennend | | O | O | |
| stechend | | O | O | |
| ziehend | | O | O | |
| wie elektrische Schläge | | O | O | |
| reißend | | O | O | |
| Anders, und zwar: | | | | |
| Weiß nicht | | O | O | |
| **12) Gibt es Auslöser für Ihre Schmerzen? Hier können Sie mehrere Antworten ankreuzen.** | | | | |
|  | Als Erwachsener | | | In der Kindheit |
| Spontane Schmerzen ohne Auslöser | O | | | O |
| Hitze | O | | | O |
| Kälte | O | | | O |
| Fieber | O | | | O |
| Körperliche Anstrengung | O | | | O |
| Sport | O | | | O |
| Andere, und zwar: | | | | |
| Weiß nicht | O | | | O |

| **13) Wie viele Arbeitsfehltage (einschließlich Hausarbeit) hatten Sie aufgrund von Schmerzen im vergangenen Jahr? Hier sind auch Wochenenden gemeint, an denen Sie beispielsweise aufgrund von Schmerzen nicht aufstehen können.** |
| --- |
| ________________ Arbeitsfehltage |

| **14) Wie sehr beeinträchtigen Schmerzen Ihre Arbeitsfähigkeit (einschließlich Hausarbeit) generell auf einer Skala von null bis zehn? Null bedeutet dabei „keine Beeinträchtigung“ und zehn „Arbeit ist unmöglich“.** | |
| --- | --- |
|  | Bitte nur eine Zahl ankreuzen |
| 0 – 1 – 2 – 3 – 4 – 5 – 6 – 7 – 8 – 9 – 10  gar Arbeit  nicht beeinträchtigt unmöglich | |

| **15) Wie sehr beeinträchtigen Schmerzen Ihre Freizeitaktivitäten oder Unternehmungen generell? Null bedeutet dabei „keine Beeinträchtigung“ und zehn „Freizeitaktivitäten sind unmöglich“.** | |
| --- | --- |
|  | Bitte nur eine Zahl ankreuzen |
| 0 – 1 – 2 – 3 – 4 – 5 – 6 – 7 – 8 – 9 – 10  gar Freizeitaktivitäten  nicht beeinträchtigt unmöglich | |
